# The Use of Ultrasound-Guided 3D-Constructed Obturator Device in the Management of Cleft Lip and Palate: A Case Series

**DOI:** 10.7759/cureus.64948

**Published:** 2024-07-19

**Authors:** Varun Muddasani, Santosh Kumar Kamalakannan, Harish S, Asha Arun, Kumutha J

**Affiliations:** 1 Pediatrics and Neonatology, Saveetha Medical College and Hospitals, Saveetha Institute of Medical and Technical Sciences, Saveetha University, Chennai, IND

**Keywords:** rehabilitation, nutritional recovery, 3d printing, ultrasound-guided, 3d obturator device, craniofacial anomalies

## Abstract

Oral clefts represent a significant craniofacial anomaly in neonates, presenting multifaceted challenges such as feeding difficulties, recurrent ear infections, speech impediments, poor growth, hearing impairments, and dental misalignments. These anomalies not only affect physical health but also have profound psychosocial implications for affected individuals and their families. Current management strategies aim to address these challenges comprehensively, and recent advancements in technology have offered innovative solutions. Among these, the integration of ultrasound-guided (USG) three-dimensional (3D)-constructed obturator devices has emerged as a promising approach to enhancing patient outcomes, particularly in achieving facial symmetry and facilitating early nutritional rehabilitation. This study presents a detailed case series of three term infants born to non-consanguineous parents with appropriate birth weights for their gestational age, each diagnosed with a unilateral cleft lip and palate (UCLP).

The first infant also presented with left-hand polydactyly and a preauricular sinus, while the second was diagnosed with multicystic kidney disease based on kidney, ureter, and bladder (KUB) scan findings. Collaborating with the Smile Train organization and the maxillofacial surgery team, a comprehensive management plan was devised. In the initial phase, intraoral scanning (Medit Intraoral Scanner™, Seoul, South Korea; done at Saveetha Medical College and Hospitals, Chennai) and digital printing of the obturator plate were performed to capture precise anatomical details. Subsequently, 3D printing technology (Ender 3D Printer™, Creality, Shenzhen, China; done at Saveetha Medical College and Hospitals, Chennai) was employed to fabricate a customized obturator plate equipped with a nasal stent. This ultrasound (US)-guided 3D-constructed obturator device was designed to fit each infant's unique oral anatomy, providing optimal support and alignment. The implementation of this device within a week post birth played a pivotal role in expediting the initiation of direct breastfeeding and nutritional rehabilitation. Furthermore, one of the infants underwent cleft lip surgical repair at four months of age, showcasing the device's compatibility with subsequent surgical interventions. The utilization of US-guided 3D-constructed obturator devices in the management of cleft lip and palate (CLP) has demonstrated significant clinical benefits. These devices contribute to reduced facial deformities, mitigate nasal cartilage sagging, and foster enhanced weight gain. Additionally, they facilitate successful breastfeeding, thereby promoting early nutritional recovery. Moreover, the improved facial symmetry and cheek fullness resulting from this approach contribute to accelerated rehabilitation, thereby reducing the societal stigma often associated with craniofacial anomalies.

## Introduction

Cleft lip and palate (CLP) are among the most common congenital deformities affecting the orofacial region. These anomalies arise when there is an incomplete fusion of the lip and/or palate during fetal development, resulting in a gap or cleft. This condition poses significant challenges, not only due to its physical manifestations but also because of the associated feeding difficulties and the emotional and psychological impact on families [1]. CLP occur with an incidence of approximately one in 750 live births globally, affecting around 0.133% of newborns. The condition shows a marked familial pattern, suggesting a genetic predisposition, and varies significantly among different racial groups. For instance, Asian populations tend to have higher rates of CLP compared to African populations, which exhibit the lowest incidence. Gender also plays a crucial role, with males being twice as likely to be affected by CLP as females. This disparity could be due to the different patterns of genetic expression and hormonal influences during fetal development [2,3]. In India, the prevalence of CLP ranges from 0.25 to 1.56 per 1,000 live births. This variation can be attributed to diverse genetic backgrounds, environmental factors, and access to healthcare services across different regions. The higher end of this prevalence range indicates a significant public health concern, necessitating targeted interventions and support systems for affected families [4].

One of the most immediate and life-threatening challenges faced by infants with CLP is difficulty in feeding. The abnormal communication between the oral and nasal cavities impedes the infant's ability to create the necessary suction for effective breastfeeding or bottle-feeding. Common feeding problems include nasal regurgitation, where milk flows back through the nose; poor suction leading to the inadequate intake of milk; increased air intake resulting in frequent burping; and prolonged feeding times. These difficulties can lead to poor weight gain and failure to thrive if not adequately managed [5]. Feeding a newborn with a cleft lip alone, as opposed to those with both cleft lip and palate, can be similar to feeding a non-affected infant, provided there are no other systemic issues. However, even in these cases, ensuring proper nutrition remains a significant concern due to the potential for feeding difficulties. The effective management of these challenges is crucial for the infant's growth and development, as well as for alleviating the stress and anxiety experienced by parents [6]. Common feeding problems experienced by cleft patients include nasal regurgitation, poor suction, increased air intake, recurrent burping, and longer feeding duration [7].

To address the feeding difficulties faced by infants with CLP, various prosthetic and supportive interventions have been developed. One such intervention is the use of flexible feeding plates, also known as obturators. These prosthetic devices help to cover the cleft in the palate, creating a more normal separation between the oral and nasal cavities. This separation enables the infant to create better suction and reduces the incidence of nasal regurgitation and air intake during feeding. The use of feeding obturators is typically considered a temporary measure until the surgical repair of the cleft can be performed. The surgical closure of the cleft lip and/or palate is usually done in stages, with initial surgeries often taking place within the first few months of life, depending on the infant's health and growth. In recent years, advances in medical technology have led to the development of new techniques such as intrauterine fetal cleft closure using an endoscopic approach. Similarly, three-dimensional (3D) printing of such obturator devices helps in achieving early rehabilitation. Three-dimensional (3D) printing is an additive manufacturing technique that prints small layers of material and then fuses them together to form a physical product from a digital design. Using bespoke scans, some companies, such as those that make cars, airplanes, and hearing aids, use 3D printing to construct prototypes and mass-produce their products.

Even though it is now too sluggish to be used in mass production, this technique, still in the experimental stages, offers the potential for correcting the cleft before birth, thereby eliminating the associated feeding challenges from the outset [8]. Ensuring proper nourishment for infants with CLP is essential for their growth and development. Nutritional management should begin immediately after birth, with the introduction of feeding obturators and the use of specialized feeding techniques. Healthcare providers, including pediatricians, nutritionists, and lactation consultants, should work closely with parents to develop a feeding plan tailored to the infant's needs. Monitoring the infant's weight gain and growth parameters is crucial. Regular assessments help to identify any deficiencies early and allow for timely interventions [9]. This case series discusses the use of ultrasound-guided (USG) 3D printing to create obturator plates for babies with cleft lip and palate (CLP). This innovative approach enabled the early establishment of adequate feeding, which supported the babies' growth, facilitated earlier surgical procedures, and promoted quicker rehabilitation.

## Case presentation

**Figure 1:**
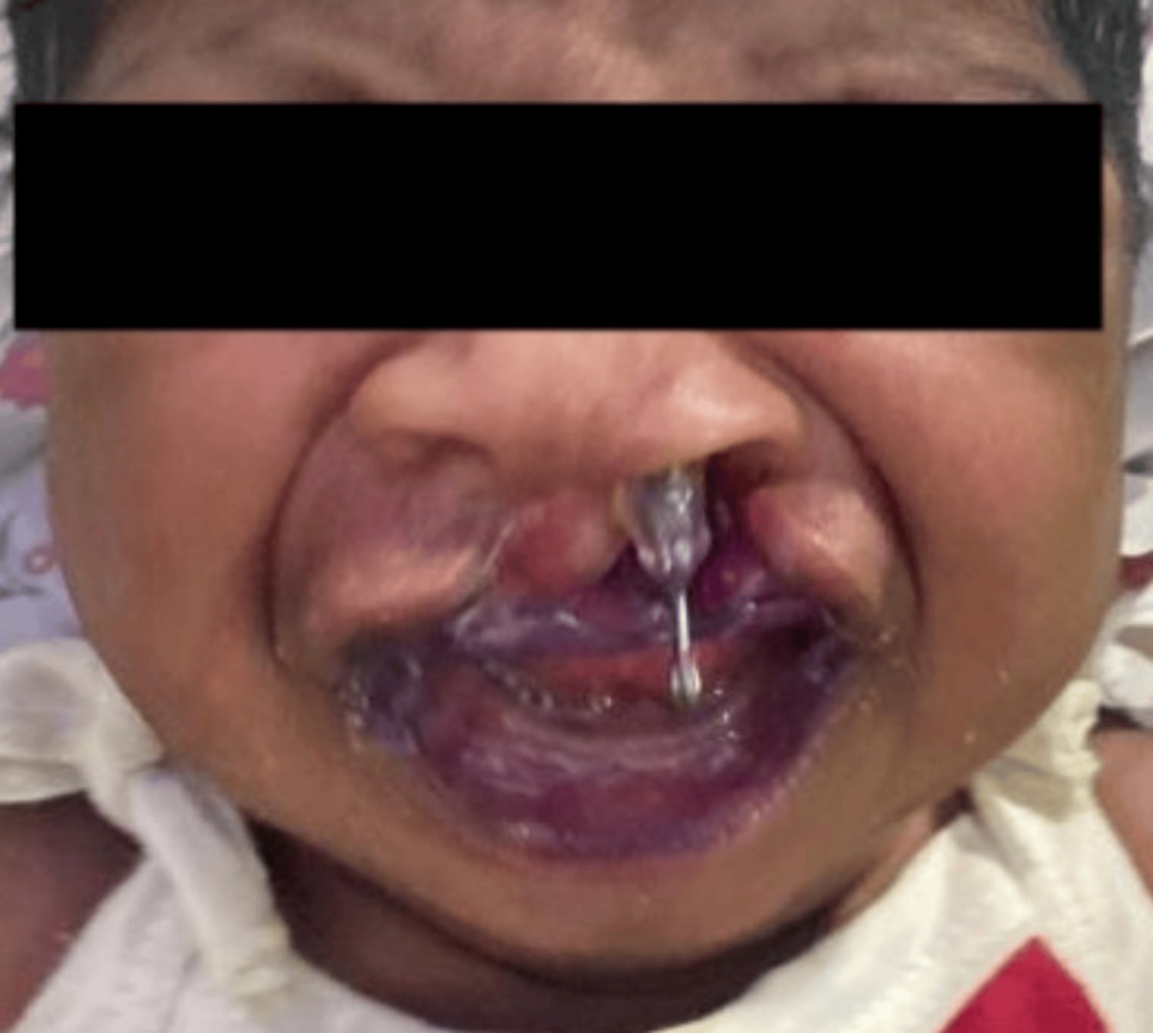
Baby with cleft lip and palate with the obturator plate and nasal stent in situ

**Figure 2:**
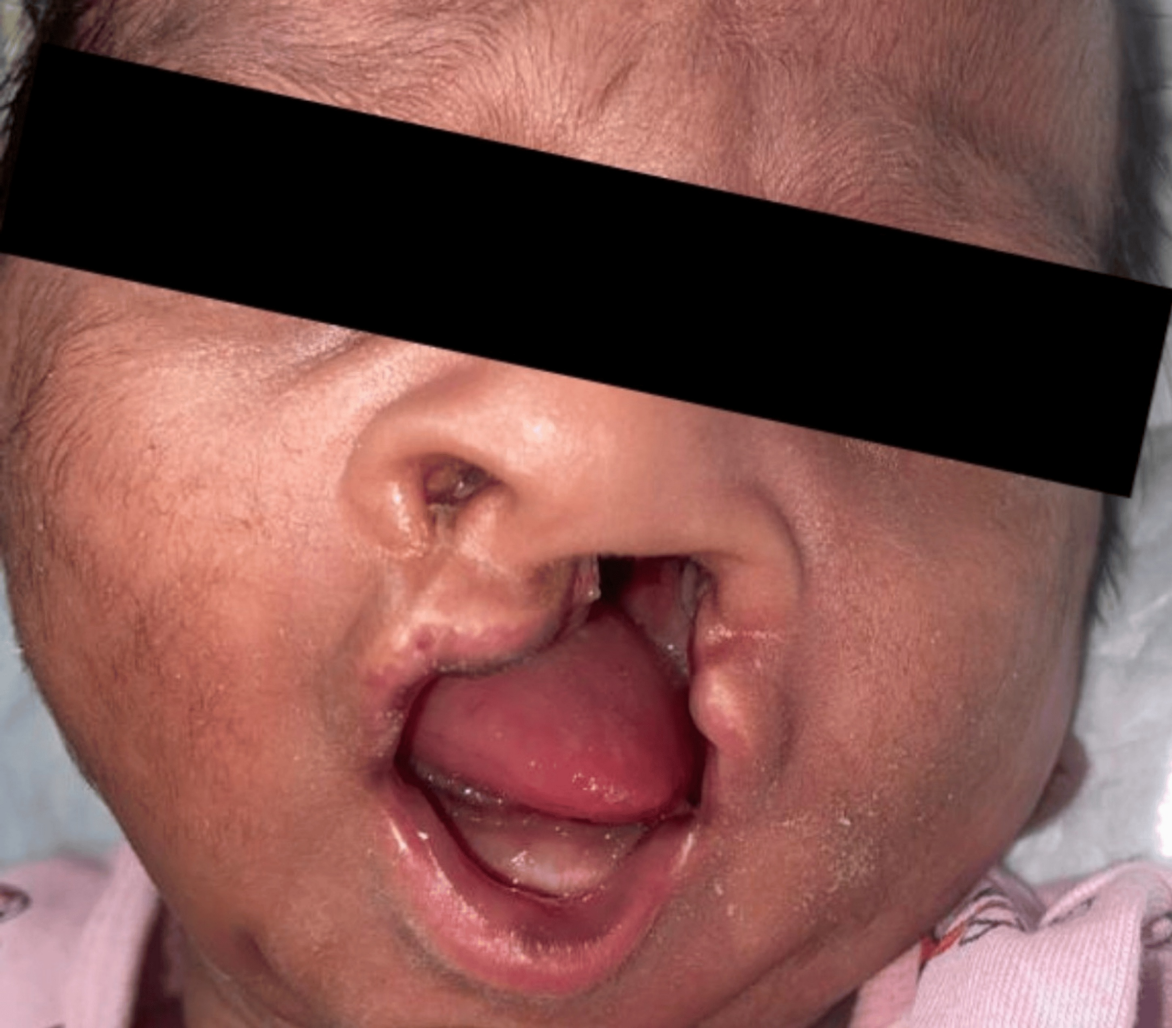
Baby from the case series with the mentioned cleft lip and palate, with an additional deformity of polydactyly of the left hand and preauricular sinus

**Figure 3:**
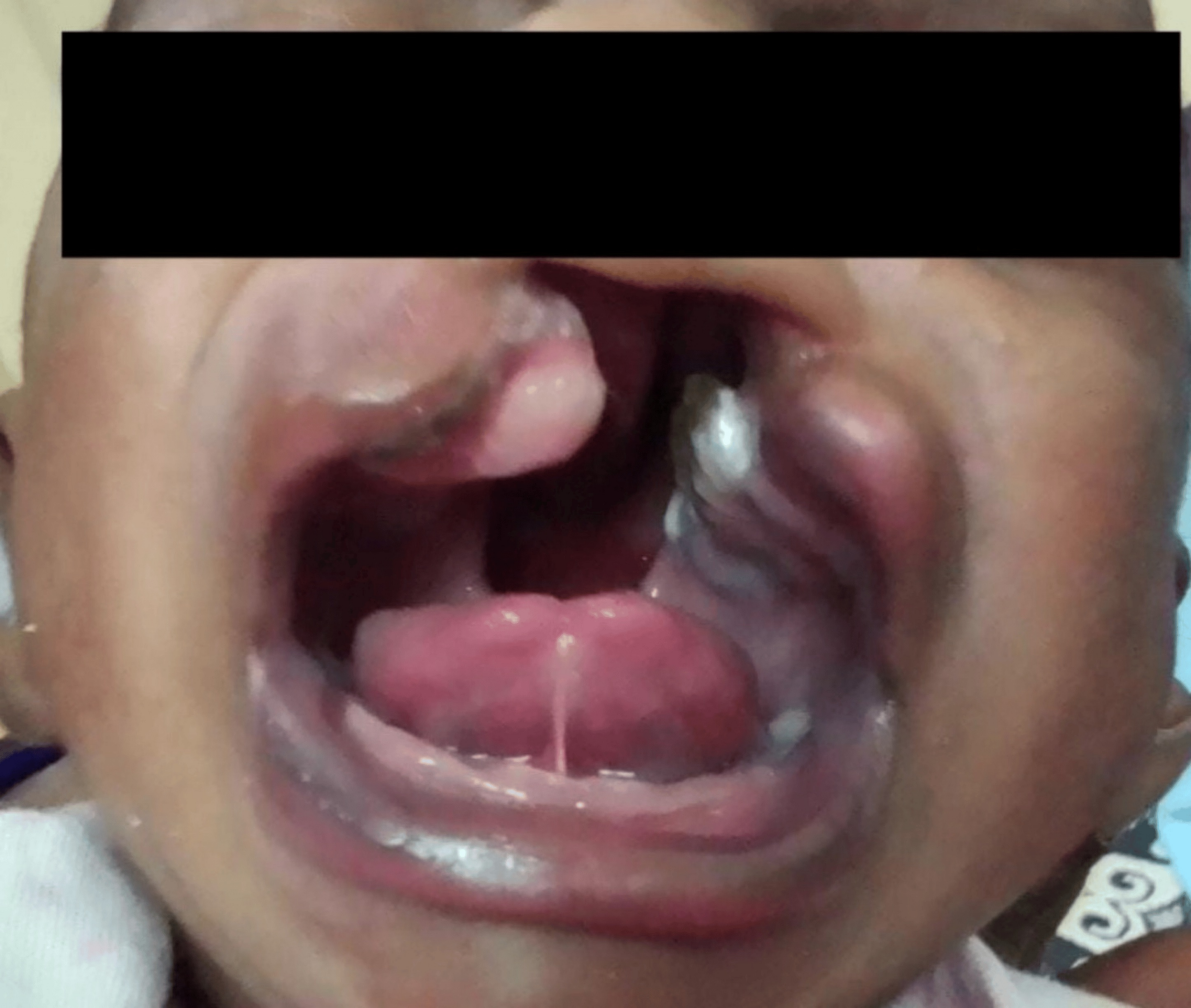
Baby with cleft lip and palate with additional multicystic kidney disease

Here, we present case details of three term babies (Figure 1) who were born out of non-consanguineous marriage with appropriate birth weight for gestational age. They had unilateral cleft lip and palate (UCLP). One baby had an additional deformity of polydactyly of the left hand and preauricular sinus (Figure 2). The second baby showed multicystic kidney disease on a kidney, ureter, and bladder (KUB) scan (Figure 3).

**Figure 4:**
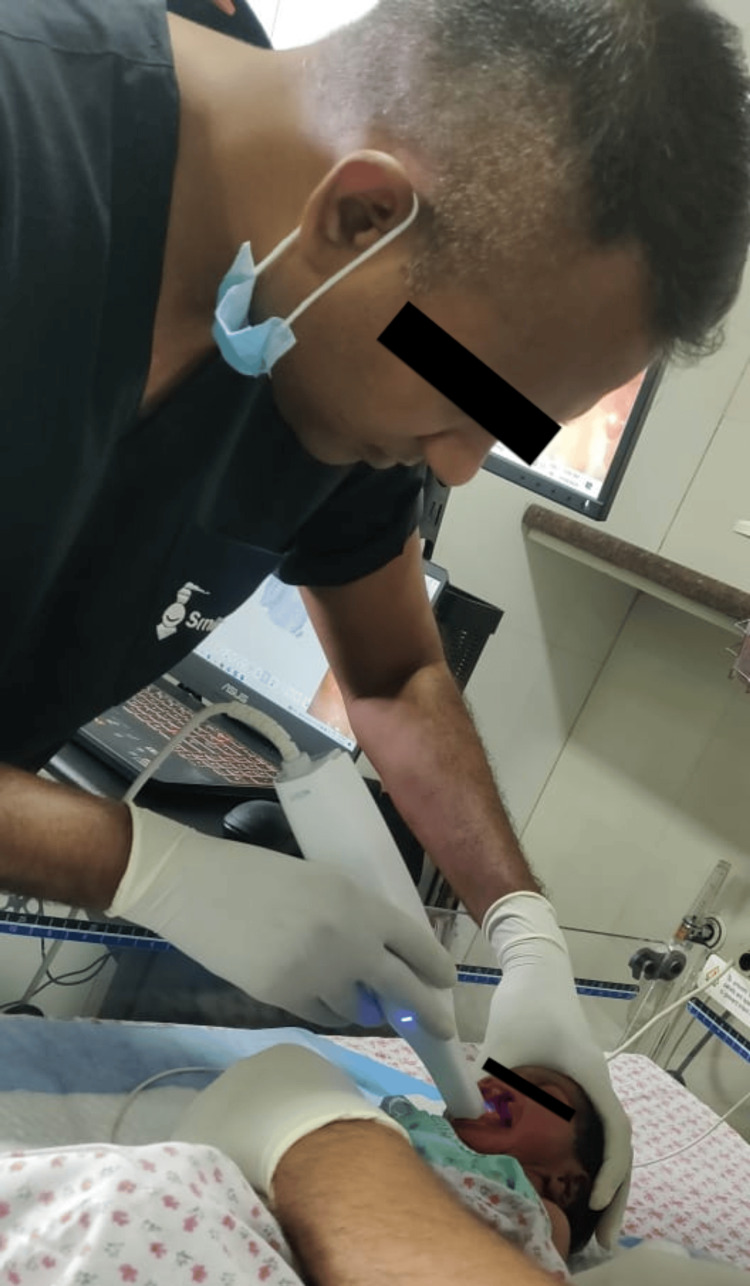
A doctor from the Smile Train organization performing the intraoral scan for designing (Medit Intraoral Scanner™, Seoul, South Korea; done at Saveetha Medical College and Hospitals, Chennai) and digital printing the obturator plate (Ender 3D Printer™, Creality, Shenzhen, China; done at Saveetha Medical College and Hospitals, Chennai)

**Figure 5:**
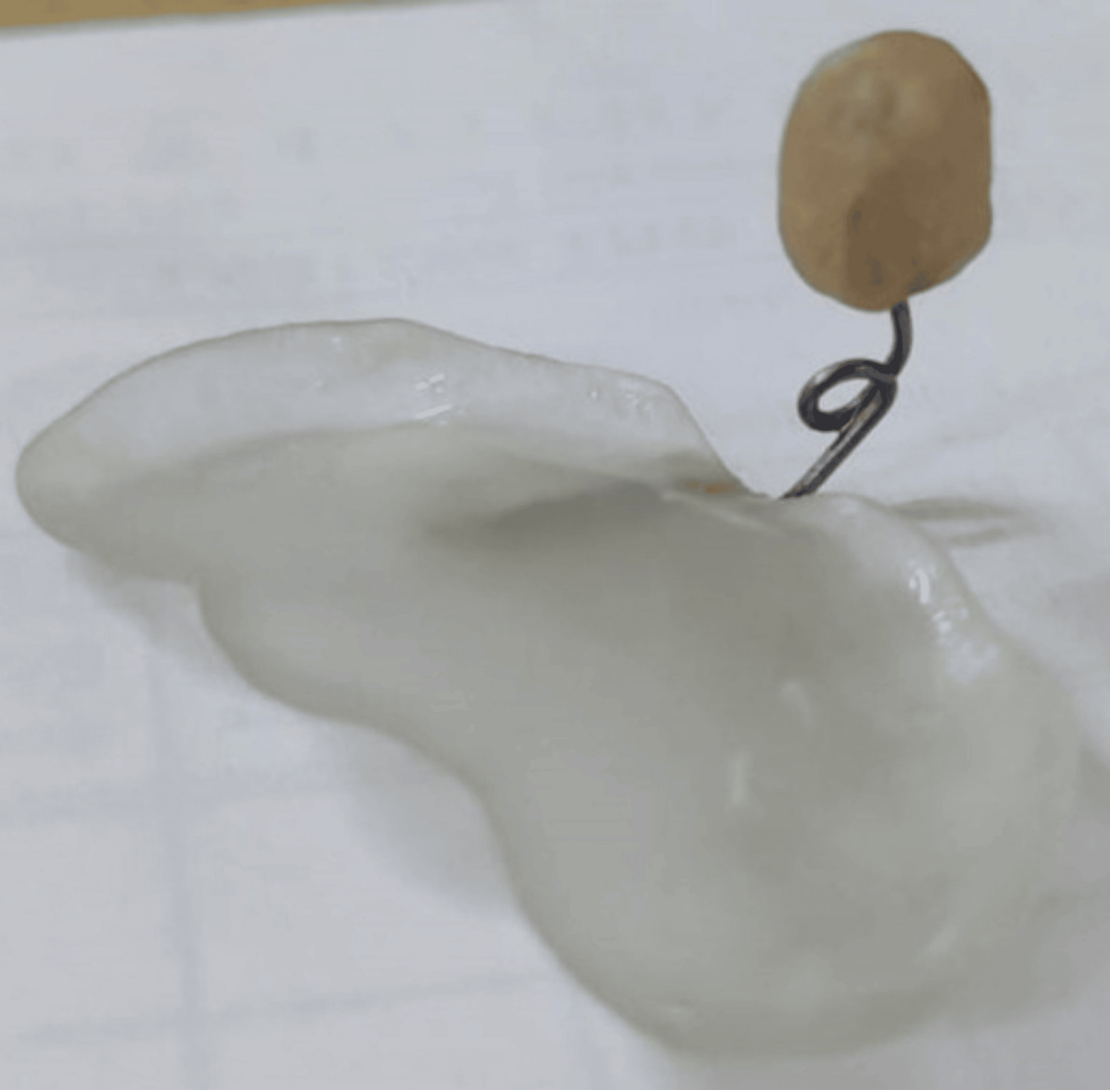
Obturator plate with a nasal stent made using 3D printing technology (Ender 3D Printer™, Creality, Shenzhen, China; done at Saveetha Medical College and Hospitals, Chennai) 3D: three-dimensional

**Figure 6:**
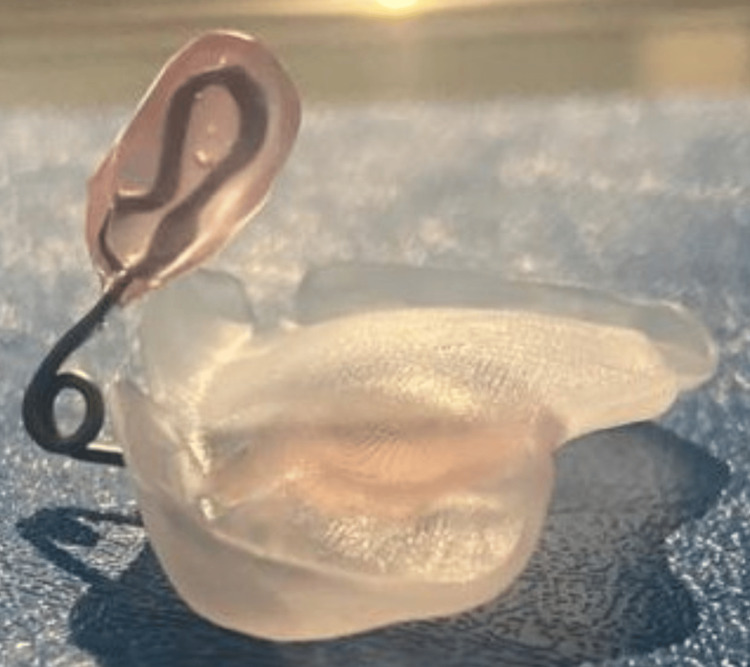
Nasal strut for elevating the nasal dome and also supporting the nasal cartilage (Ender 3D Printer™, Creality, Shenzhen, China; done at Saveetha Medical College and Hospitals, Chennai)

**Figure 7:**
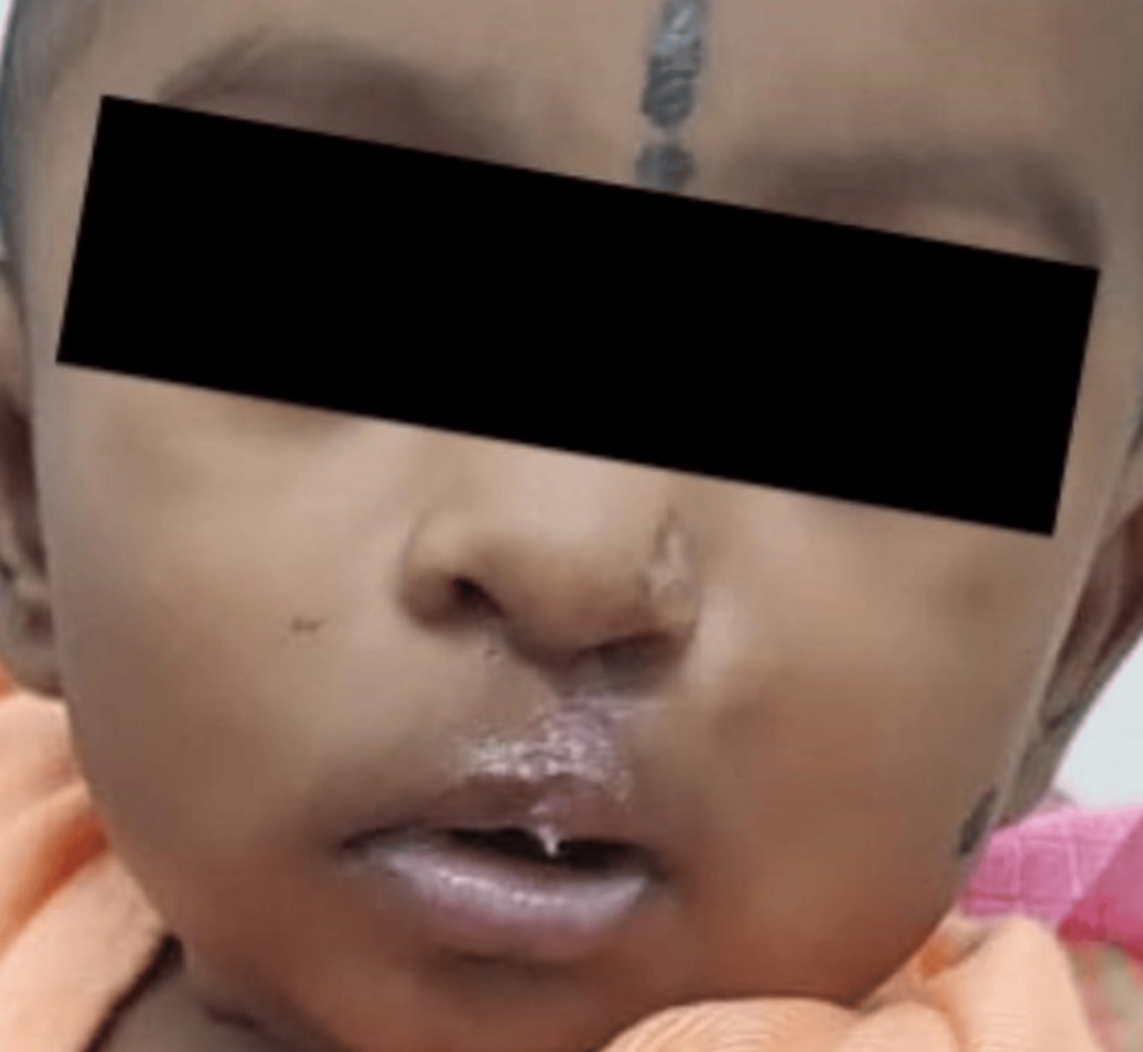
Postoperative image of the baby at four months of age who retained the obturator plate for three months prior to corrective surgery

**Figure 8:**
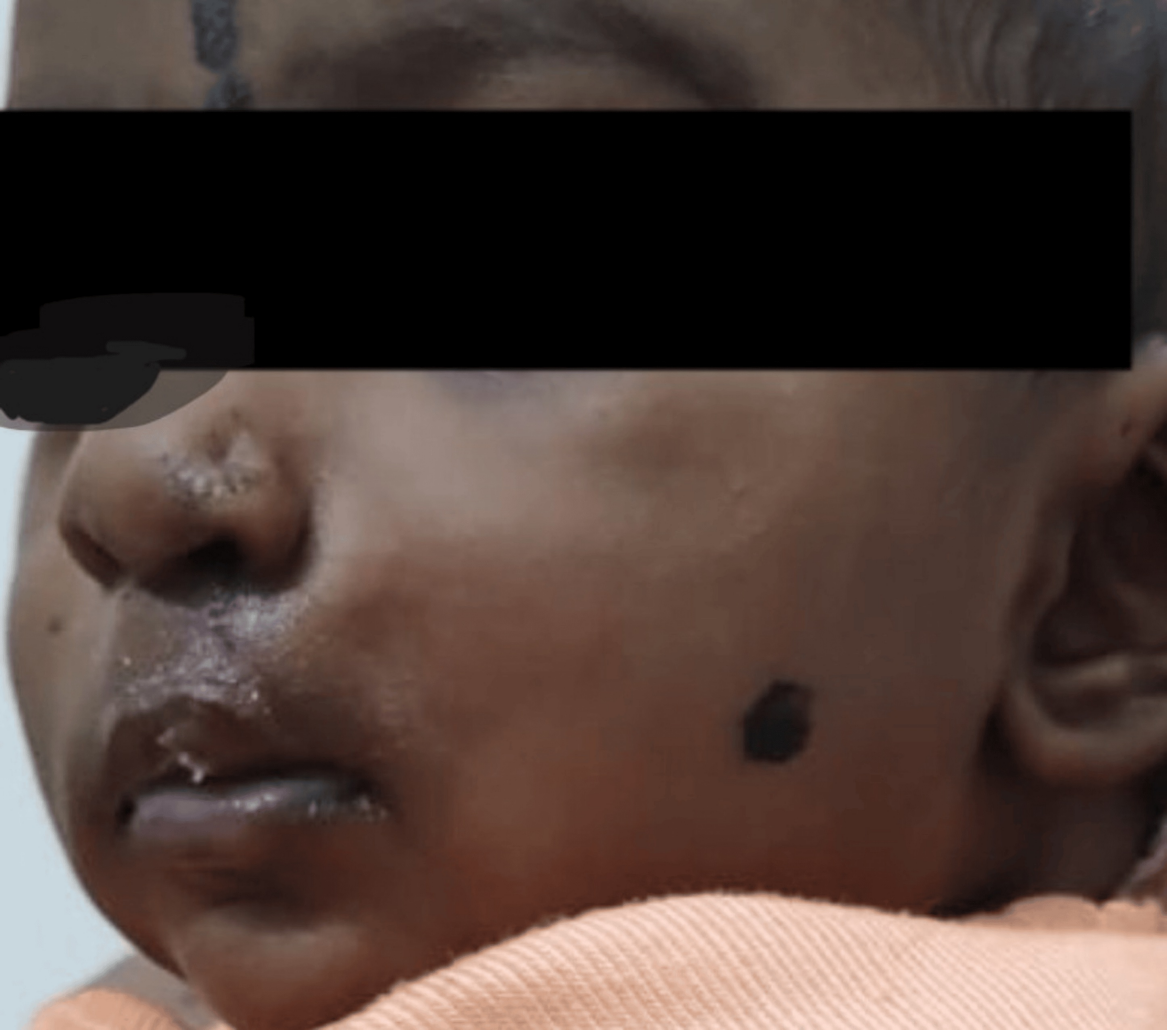
The obturator plate with nasal strut elevated the nasal dome and retained cartilage shape helping preserve the facial symmetry

In collaboration with the Smile Train organization and maxillofacial surgery team, intraoral scanning is performed using Medit Intraoral Scanner™ (Seoul, South Korea; done at Saveetha Medical College and Hospitals, Chennai), and the 3D printing of obturator plate with a nasal stent (Ender 3D Printer™, Creality, Shenzhen, China; done at Saveetha Medical College and Hospitals, Chennai) was done as seen in Figure 4. A 3D-constructed obturator plate with a nasal stent was devised for use within a week from birth (Figures 5, 6). It helped in the quicker establishment of direct breastfeeding and nutritional rehabilitation. One of the babies underwent a surgical repair of cleft lip at four months of age as seen in Figure 7 and Figure 8.

## Discussion

Presurgical infant orthopedics (PSIO) has a long history, dating back to 1686, with the aim of improving nose aesthetics and reducing the cleft gap width before surgical lip closure [10]. The introduction of nasoalveolar molding (NAM) by Figueroa and Polley in 1993 marked a significant advancement, utilizing an intraoral plate with a nasal stent attached for future adjustments [11]. Subsequent developments in digital orthodontics led to the introduction of computer-aided design/nasoalveolar molding (CAD/NAM) by Yu et al. in 2011, where a series of 3D-printed models were used to construct appliances for each patient [12,13]. In a randomized controlled trial conducted by Abd El-Ghafour et al., 34 nonsyndromic infants with UCLP were assigned to either a no-treatment (control) group or the D-NAM group. The D-NAM group underwent a procedure where maxillary models were 3D-scanned and segmented, followed by an approximation of alveolar segments and the construction of a virtual appliance, which was then 3D-printed. Nasal stents were added to the appliances, and horizontal tapes were applied. Blinded assessors measured maxillary arch dimensions before and after treatment, revealing significant improvements in the D-NAM group compared to the control group [14]. This was also underscored in a case report by Bansal et al., which detailed the management of a one-day-old neonate with complete bilateral cleft lip and palate. The report emphasized the pivotal role of palatal obturators in assisting feeding, as well as promoting speech and language development and presurgical orthopedics and mitigating associated otorhinolaryngeal issues [15]. In a recent study by Patel et al. [16], the feasibility and accuracy of utilizing digital intraoral scanning technology for infants diagnosed with cleft lip and palate (CLP) were investigated, comparing its efficacy to conventional alginate impressions. The primary aim was to assess whether digital impressions offer safer and more precise results. The research centered around a three-month-old infant diagnosed with bilateral CLP. The study findings revealed that digital intraoral scanning presents a viable alternative to traditional impression techniques. Notably, digital scans were observed to be faster, more accurate, and safer, with a reduced risk of foreign body dislodgement and airway obstruction. This study underscored the potential benefits of digital technology in enhancing the treatment planning and documentation process for infants with CLP [16].

A case report by Ijaz et al. [17] discusses an innovative modification to the nasoalveolar molding plate for the presurgical treatment of a neonate with unilateral cleft lip and palate. The modification included adding an acrylic stent to the existing self-retentive plate, along with wire stents for nasal molding. This device was applied to a three-day-old neonate with a left-sided cleft lip and palate. The addition of a wire nasal stent to position under the tip of the nose elevated the nasal dome and directed the cartilage toward the nasal tip. Weekly activations were performed until the age of three months. Photocopy analysis revealed a significant reduction of 6 mm in the anterior cleft region, an elevation of the alar tip and nasal alar dome, and improved symmetry before cheiloplasty. The modified device effectively approximated the major alveolar segment, resulting in minimized lip scar and a pleasant facial appearance. Studies have demonstrated that digital impression systems yield similar precision and greater reproducibility compared to conventional techniques for recording dental hard tissues [18-20].

The use of ultrasound-guided 3D printing of obturator plates for neonates with cleft lip and palate (CLP) represents a significant advancement in the field, offering improved feeding outcomes and potentially enhancing early surgical interventions and overall rehabilitation. However, several limitations must be considered. The technical complexity of creating these plates requires specialized equipment and expertise in both ultrasound imaging and 3D printing technology, limiting availability to specialized centers. The advanced technology is expensive, potentially making it less accessible for families in low-resource settings or countries with limited healthcare funding. Implementing this technology necessitates training healthcare providers in ultrasound imaging, 3D printing software, and the creation of obturator plates, which can be time-consuming and require significant investment. Variability in ultrasound imaging quality, influenced by the operator's skill and equipment, can lead to less accurate 3D models and less effective obturator plates. The process of obtaining ultrasound images, designing the 3D model, and printing the obturator plate can be time-consuming, posing a problem in cases needing immediate intervention. Rapid growth in neonates may necessitate frequent adjustments or replacements of the obturator plate to ensure proper fit and comfort, and the initial fitting process may involve trial and error. While promising, this technique's widespread clinical validation is still in its early stages, requiring more extensive studies and long-term follow-ups to fully establish its efficacy and safety. Errors in the imaging, design, or printing process can lead to poorly fitting obturator plates, causing discomfort, interfering with feeding, or failing to achieve desired therapeutic outcomes. This technology is primarily designed for CLP cases and may not be suitable for other craniofacial anomalies or more complex congenital conditions. Finally, the success of using an obturator plate depends on parental acceptance and compliance, including regular visits for adjustments and careful handling of the device. Parental anxiety and the lack of understanding might pose challenges. Despite these limitations, further research and development can help address these issues and expand the accessibility and efficacy of this innovative approach.

## Conclusions

The rehabilitation journey of children with CLP typically commences at birth, often involving oral impressions as one of the initial procedures conducted to document the cleft and arch form or fabricate oral appliances. However, conventional impression-taking in neonates is a technique-sensitive process fraught with elevated risks compared to older infants. The advent of digital dental technologies and intraoral optical range-finding scanning offers the potential for safer, more effective, and more accurate impressions in infants with CLP. The successful rehabilitation of cleft lip and palate in neonates in this case series has been achieved through the use of an ultrasound-guided 3D-constructed obturator device. These appliances alleviate parental concerns and provide infants with efficient suction around the nipple, improving feeding efficiency.
